# Circulating DNA and Neutrophil-Derived Biomarkers in Neonatal Sepsis

**DOI:** 10.3390/ijms27104500

**Published:** 2026-05-18

**Authors:** Ana Maria Behrami, Jasmin Knopf, Michael Boettcher, Chinedu Ulrich Ebenebe

**Affiliations:** 1 Department of Pediatrics, University Medical Center Hamburg, 20246 Hamburg, Germanychinedu.ebenebe@st-marienkrankenhaus.de (C.U.E.); 2 Department of Pediatrics, Friedrich-Ebert-Krankenhaus, 24534 Neumünster, Germany; 3 Department of Pediatric Surgery, University Medical Center Mannheim, Heidelberg University, 68167 Mannheim, Germany; jasmin.knopf@medma.uni-heidelberg.de; 4 Mannheim Institute for Innate Immunoscience (MI3), Medical Faculty Mannheim, Heidelberg University, 68167 Mannheim, Germany; 5 Mannheim Center for Inflammation Medicine (MaCIM), Medical Faculty Mannheim, Heidelberg University, 68167 Mannheim, Germany; 6 Department of Pediatrics, St. Marienkrankenhaus, 67067 Ludwigshafen, Germany

**Keywords:** neonatal sepsis, neutrophil extracellular traps, cell-free DNA, myeloperoxidase–DNA complexes, citrullinated histone H3, biomarkers

## Abstract

Neutrophil extracellular traps (NETs) contribute to innate immunity in sepsis, but their diagnostic value in neonates is unclear. We evaluated whether circulating NET-associated biomarkers discriminate septic from non-infected neonates. In this prospective observational study 96 neonates (≥34 weeks gestational age) with clinical suspicion of infection were enrolled (36 sepsis, 60 controls). Serum cell-free DNA (cfDNA), myeloperoxidase–DNA complexes (MPO-DNA), neutrophil elastase–DNA complexes (NE-DNA), and citrullinated histone H3 (H3cit) were measured alongside CRP and IL-6 at days 1, 3, and 5. Diagnostic performance was assessed by receiver operating characteristic (ROC) analysis with bootstrap confidence intervals. CRP (AUC 0.75, 95% CI 0.66–0.85) and IL-6 (AUC 0.73, 95% CI 0.61–0.83) showed the best diagnostic performance. cfDNA demonstrated moderate discrimination (AUC 0.72, 95% CI 0.60–0.84) but was only transiently elevated at day 1. MPO-DNA (AUC 0.47), NE-DNA (AUC 0.44), and H3cit (AUC 0.47) performed no better than chance. Within the sepsis group, MPO-DNA and NE-DNA at day 3 strongly correlated with the immature-to-total neutrophil ratio (ρ = 0.76 and 0.72), suggesting these markers reflect neutrophil degranulation rather than NET formation. NET-associated biomarkers do not improve diagnostic accuracy for neonatal sepsis beyond CRP and IL-6. These findings support the concept that neonatal innate immune responses differ fundamentally from adult patterns.

## 1. Introduction

Neonatal sepsis is a life-threatening bloodstream infection affecting infants younger than 28 days of life and is conventionally classified as early-onset (≤72 h after birth) or late-onset (>72 h after birth) [1]. Recent multicentre data confirm that sepsis remains a major contributor to morbidity and mortality across the paediatric age range, including the neonatal period [2]. Neonatal sepsis remains a leading cause of morbidity and mortality in the neonatal period, affecting an estimated 3 million neonates worldwide annually and contributing to approximately one-quarter of all neonatal deaths [3,4]. Early diagnosis is critical for timely intervention, yet it is hampered by non-specific clinical signs that overlap with non-infectious conditions such as respiratory distress syndrome and perinatal asphyxia. Currently used biomarkers, including C-reactive protein (CRP) and interleukin-6 (IL-6), have well-recognized limitations: CRP rises with a delay of 12–24 h after infection onset, while IL-6 peaks early but declines rapidly, resulting in a narrow diagnostic window [3,4]. Blood cultures, the reference standard, are positive in only a minority of clinically diagnosed cases and require prolonged incubation times. Consequently, there is an urgent need for novel biomarker strategies that enable earlier and more reliable identification of neonatal sepsis.

Neutrophils are short-lived innate immune cells that act as the first cellular line of host defence and are often regarded as the foot soldiers of the immune response, deploying phagocytosis, granule release and the formation of extracellular traps to attack bacteria, viruses and fungi. Neutrophil extracellular traps (NETs) are web-like extracellular chromatin structures decorated with antimicrobial proteins such as myeloperoxidase (MPO), neutrophil elastase (NE), and histones [5]. In adult sepsis, circulating NET components have been shown to correlate with disease severity, organ dysfunction, and mortality [6,7,8]. However, neonatal neutrophils exhibit significant developmental differences, including reduced NADPH oxidase activity, diminished reactive oxygen species (ROS) production, and delayed NET formation capacity [9,10,11]. These maturational constraints raise fundamental questions about whether NET-derived biomarkers can serve as reliable diagnostic indicators in neonates. To date, no study has systematically evaluated the diagnostic performance of a comprehensive panel of NET-associated markers against established biomarkers in a prospective neonatal cohort. We therefore aimed to compare the diagnostic accuracy of NET markers with CRP and IL-6 for discriminating neonatal sepsis from non-infectious conditions, and to characterize the temporal kinetics and inter-marker correlations of these biomarkers.

## 2. Results

Ninety-six neonates were enrolled: 36 with sepsis (30 early-onset sepsis [EOS], 6 late-onset sepsis [LOS]) and 60 non-infected controls (54 EOS workup, 6 LOS workup). Baseline characteristics were comparable between groups: median birth weight was 3292 g (IQR 2715–3590) in the sepsis group versus 2982 g (IQR 2480–3520) in controls (*p* = 0.21); median gestational age was 39 weeks (IQR 37–40) versus 38 weeks (IQR 36–40; *p* = 0.35). Sex distribution did not differ significantly (58% male in sepsis vs. 52% in controls; *p* = 0.54). Leukocyte counts at presentation were comparable (*p* = 0.41). CRP and IL-6 were significantly elevated in the sepsis group at presentation (both *p* < 0.001). Blood cultures were positive in four cases (11.1%), all in the LOS subgroup (Staphylococcus epidermidis, n = 2; Escherichia coli, n = 1; group B Streptococcus, n = 1).

**Figure 1:**
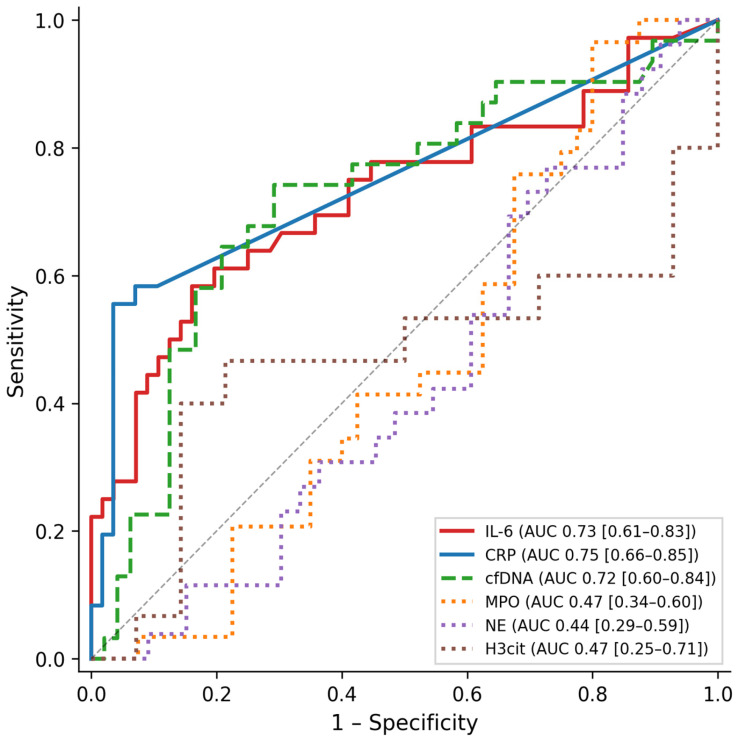
Receiver operating characteristic (ROC) curves for day 1 biomarkers discriminating neonatal sepsis from controls. Solid lines: established markers (CRP, IL-6); dashed line: cfDNA; dotted lines: NET-specific markers (MPO-DNA, NE-DNA, H3cit). AUC = area under the curve; CI = confidence interval.

At day 1, cfDNA was significantly higher in the sepsis group compared with controls (median 716 vs. 575 ng/mL; *p* = 0.001; rank-biserial r = 0.42), representing a moderate effect size. This difference resolved by day 3 (median 621 vs. 584 ng/mL; *p* = 0.31) and remained non-significant at day 5 (*p* = 0.44), indicating a transient early elevation pattern. In contrast, MPO-DNA, NE-DNA, and H3cit did not differ between sepsis and control groups at any time point (all *p* > 0.15). CRP and IL-6 showed highly significant differences at all three time points (all *p* < 0.001), with CRP peaking at day 3 and IL-6 showing the largest difference at day 1 (Figure 1).

ROC analysis of day 1 biomarker concentrations (Table 1; Figure 1) demonstrated the best diagnostic performance for CRP (AUC 0.75, 95% CI 0.66–0.85; sensitivity 56%, specificity 96% at optimal cutoff 11.0 mg/L) and IL-6 (AUC 0.73, 95% CI 0.61–0.83; sensitivity 58%, specificity 84% at cutoff 189.7 ng/L). cfDNA showed moderate discrimination (AUC 0.72, 95% CI 0.60–0.84; sensitivity 74%, specificity 71% at cutoff 653.1 ng/mL). Notably, the AUC confidence intervals of CRP, IL-6, and cfDNA overlapped considerably, suggesting comparable diagnostic performance among these three markers. In contrast, MPO-DNA (AUC 0.47, 95% CI 0.34–0.60), NE-DNA (AUC 0.44, 95% CI 0.29–0.59), and H3cit (AUC 0.47, 95% CI 0.25–0.71) performed no better than chance, with AUC values indicating no discriminatory capacity.

Correlation analysis within the sepsis subgroup revealed that MPO-DNA and NE-DNA at day 3 were strongly correlated with the immature-to-total neutrophil (I/T) ratio (ρ = 0.76 and 0.72, respectively) and immature neutrophil percentage (ρ = 0.73 and 0.69; all *p* < 0.01). MPO-DNA and NE-DNA were highly intercorrelated (ρ = 0.92, *p* < 0.001), indicating biological redundancy between these markers. These correlations were not observed in the control group, suggesting that MPO-DNA and NE-DNA reflect infection-driven neutrophil activation. cfDNA correlated weakly with CRP at day 1 (ρ = 0.29, *p* = 0.01) but showed no significant correlation with MPO-DNA (ρ = 0.11, *p* = 0.42), NE-DNA (ρ = 0.08, *p* = 0.58), or H3cit (ρ = 0.14, *p* = 0.51), reinforcing the interpretation that cfDNA elevation in neonatal sepsis is driven by mechanisms other than NET formation.

## 3. Discussion

In this prospective observational study, we systematically evaluated the diagnostic performance of a comprehensive panel of NET-associated biomarkers in neonatal sepsis. Our principal finding is that classical NET-specific markers (MPO-DNA, NE-DNA, H3cit) did not discriminate between septic and non-infected neonates, with AUC values at or below the level of chance. In contrast, the established inflammatory markers CRP and IL-6 demonstrated robust, albeit moderate, diagnostic performance. cfDNA showed a transient early elevation in sepsis but lacked sustained discriminatory capacity and did not correlate with NET-specific markers, suggesting that its source in neonates is predominantly non-NETotic.

The failure of MPO-DNA and NE-DNA to discriminate sepsis from controls contrasts sharply with findings in adult sepsis, where these markers correlate with disease severity and organ dysfunction [6,7,8]. This discrepancy likely reflects fundamental developmental differences in neonatal neutrophil biology. Neonatal neutrophils exhibit reduced expression and activity of NADPH oxidase, the enzyme complex essential for ROS-dependent (suicidal) NET formation, resulting in delayed and quantitatively diminished NET formation [9,10,11]. Additionally, neonatal neutrophils show impaired chemotaxis, reduced adhesion molecule expression, and altered granule protein content, all of which may contribute to a blunted NET response during infection [12]. The strong correlation of MPO-DNA and NE-DNA with the I/T ratio and immature neutrophil percentage—observed exclusively in the sepsis group—provides important mechanistic insight: these markers likely reflect neutrophil degranulation and activation during the hematologic stress response rather than true NET formation. Their high intercorrelation (ρ = 0.92) further supports biological redundancy and a shared origin from neutrophil granule release rather than independent NET-specific processes.

The moderate discriminatory performance of cfDNA (AUC 0.72) and its transient elevation at day 1 merit careful interpretation. cfDNA originates from multiple cellular processes, including apoptosis, necrosis, pyroptosis, and physiological cell turnover, and is therefore a non-specific marker of cellular injury rather than a NET-specific indicator [13]. In neonates, additional sources of cfDNA include placental-derived fetal DNA, which undergoes rapid clearance after birth, and birth-related tissue stress [14]. The absence of correlations between cfDNA and the NET-specific markers MPO-DNA, NE-DNA, and H3cit in our cohort strongly supports the interpretation that cfDNA elevation in neonatal sepsis reflects general cellular damage associated with the inflammatory response rather than NET formation. The weak correlation with CRP (ρ = 0.29) further suggests a shared association with systemic inflammation but distinct biological origins.

Our findings are consistent with previous studies reporting limited NET formation capacity in neonates and reduced diagnostic value of NET markers in early-onset sepsis [1,11,15]. In a previous study, we demonstrated that NET formation markers did not differentiate EOS from non-infected neonates in a similar cohort, although cfDNA showed a trend toward elevation [1]. Hashem et al. reported elevated cfDNA and MPO-DNA in neonatal sepsis; however, their study included a higher proportion of preterm and culture-positive cases, which may explain the discrepancy [16]. Importantly, our study extends these observations by evaluating H3cit—the most specific NET marker available—which showed no group differences, and by demonstrating the temporal kinetics of biomarker concentrations over five days. Taken together, these results highlight that biomarker strategies validated in adult sepsis cannot be directly extrapolated to neonates and underscore the need for age-specific diagnostic approaches that account for the functional immaturity of neonatal innate immunity.

Several limitations warrant consideration. First, the relatively small sample size, particularly for LOS (n = 6 per group) and culture-positive cases (n = 4), limits the generalizability of our findings and precluded meaningful subgroup analyses for these categories. Second, the relatively small cohort, the limited number of LOS and culture-positive cases, and the use of serum rather than plasma for NET marker quantification. However, neonates are a particularly vulnerable population with restricted blood sample availability. Coagulation in serum tubes can induce ex vivo neutrophil activation, degranulation and partial NET-like processes, which may artefactually elevate cfDNA, MPO–DNA, NE–DNA and even H3cit and reduce their specificity for in vivo NETosis [17]; this preanalytical limitation likely contributes to the limited diagnostic value of MPO–DNA and NE–DNA observed here, and motivates the more neutral terminology used in the revised title. AUC values numerically below 0.5 for MPO–DNA, NE–DNA and H3cit are not biologically meaningful in this study: their 95% confidence intervals all cross 0.5, and the values are statistically indistinguishable from chance, consistent with markers carrying no discriminative information rather than truly inverse classifiers. A pre-specified combination analysis (logistic regression of NET markers added to a CRP + IL-6 baseline model) showed no improvement in AUC over CRP + IL-6 alone (ΔAUC ≤ 0.02, all 95% CIs crossing zero), confirming that NET markers do not enhance diagnostic performance beyond established biomarkers. Third, the clinical sepsis definition based on clinical signs plus CRP > 10 mg/L, while pragmatic and widely used in neonatal medicine, may have included some neonates with non-infectious inflammatory conditions and excluded culture-negative true infections with lower inflammatory responses. Finally, our cohort predominantly comprised near-term and term neonates with EOS; whether NET markers perform differently in very preterm infants or LOS warrants investigation in dedicated studies.

In conclusion, our prospective evaluation demonstrates that currently available NET-associated biomarkers—including the NET-specific markers MPO-DNA, NE-DNA, and H3cit—do not improve diagnostic discrimination for neonatal sepsis beyond established inflammatory markers such as CRP and IL-6. The strong correlation of MPO-DNA and NE-DNA with hematologic indices of neutrophil activation, rather than with NET-specific markers, suggests these complexes reflect degranulation rather than NET formation in neonates. These findings underscore the functional immaturity of neonatal innate immunity and emphasize the need for neonatal-specific biomarker strategies. Future research should focus on alternative innate immune pathways that are operational in the neonatal period, such as monocyte- and macrophage-derived inflammatory mediators, and should employ plasma-based sampling protocols to minimize ex vivo artifacts. Despite the acknowledged limitations, this study provides several novel contributions. To our knowledge, this is the first prospective neonatal cohort to systematically benchmark a comprehensive panel of NET-associated biomarkers (cfDNA, MPO–DNA, NE–DNA and the highly NET-specific H3cit) against the established markers CRP and IL-6 within the same patients, with serial sampling at days 1, 3 and 5. Three findings extend prior work: (I) H3cit—the most specific available NET marker—did not discriminate septic from non-infected neonates, providing direct evidence against an in vivo NETotic signature in this age group; (II) the high intercorrelation of MPO–DNA and NE–DNA together with their selective association with the I/T ratio in the sepsis group reframe these complexes as readouts of neutrophil degranulation rather than NET formation in neonates; and (III) cfDNA showed only a transient day-1 elevation that did not correlate with NET-specific markers, indicating a non-NETotic origin of circulating DNA in neonatal sepsis. Together these results refine the mechanistic interpretation of NET-related assays in newborns and argue against extrapolation of adult NET-based diagnostic strategies to this population.

## 4. Materials and Methods

### 4.1. Study Design and Population

This prospective observational study was conducted at the Department of Neonatology, University Medical Center Hamburg-Eppendorf (UKE), Hamburg, Germany, between January 2017 and December 2019. Neonates admitted with clinical suspicion of infection were eligible for enrollment. Inclusion criteria were: (i) gestational age ≥ 34 weeks, (ii) clinical signs suggestive of infection (respiratory distress, cardiovascular instability, temperature instability, apnea, feeding intolerance, or neurological symptoms), and (iii) initiation of empirical antibiotic therapy. Exclusion criteria included major congenital malformations, chromosomal abnormalities, and perinatal asphyxia requiring therapeutic hypothermia. Enrolled neonates were classified as sepsis or controls based on the following criteria: sepsis was defined by clinical signs of infection in combination with CRP > 10 mg/L within the first five days of the sepsis workup. Neonates who initially triggered a sepsis workup but did not fulfill the sepsis definition were classified as non-infected controls. Early-onset sepsis (EOS) was defined as onset within 72 h of life and late-onset sepsis (LOS) thereafter. The study was approved by the ethics committee of the Hamburg Medical Chamber (approval number PV5339), and written informed consent was obtained from parents or legal guardians of all participants prior to enrollment.

### 4.2. Biomarker Measurements

Venous blood samples (0.5–1.0 mL) were obtained at three predefined time points: day 1 (within 24 h of clinical suspicion), day 3 (±24 h), and day 5 (±24 h). Blood was collected into serum separator tubes, allowed to clot for 30 min at room temperature, and centrifuged at 2000× *g* for 10 min. Serum aliquots were stored at −80 °C until batch analysis. Routine clinical parameters, including CRP (immunoturbidimetry), IL-6 (electrochemiluminescence immunoassay, Cobas^®^, Roche Diagnostics), complete blood count with differential, and blood cultures (BACTEC™ Peds Plus, Becton Dickinson, Heidelberg, Germany), were measured according to standard hospital protocols. NET-associated markers were quantified from stored serum samples. Circulating cfDNA was measured using the Quant-iT™ PicoGreen™ dsDNA Assay (Thermo Fisher Scientific, Waltham, MA, USA, Cat. No. P11496). Briefly, 5 µL of serum was diluted 1:20 in TE buffer, and PicoGreen reagent was added at a 1:200 dilution. Fluorescence was measured at excitation/emission wavelengths of 480/520 nm using a microplate reader (Tecan Spark Cyto, Crailsheim, Germany). A standard curve was generated using lambda DNA (0–1000 ng/mL). MPO-DNA and NE-DNA complexes were quantified by capture ELISA as previously described [18,19]. Briefly, 96-well plates (Nunc MaxiSorp™, Roskilde, Denmark) were coated overnight at 4 °C with anti-human MPO polyclonal antibody (0.5 µg/mL; Merck, Darmstadt, Germany, Cat. No. 07-496-I) or anti-human NE monoclonal antibody (0.5 µg/mL; R&D Systems, Wiesbaden, Germany, Cat. No. MAB91673) in carbonate buffer (pH 9.6). After blocking with 3% BSA/PBS for 2 h, serum samples were incubated for 2 h at room temperature. Bound DNA was detected using a peroxidase-conjugated anti-DNA monoclonal antibody (Cell Death Detection ELISA, Roche, Mannheim, Germany, Cat. No. 11544675001) at a 1:40 dilution for 1 h. After substrate addition (TMB), absorbance was measured at 450 nm with reference at 570 nm. This sandwich ELISA design captures protein–DNA complexes released during NET formation rather than free granule proteins; however, potential limitations regarding in vivo NET specificity of the MPO-DNA ELISA have been discussed [17]. H3cit was measured using the Citrullinated Histone H3 (Clone 11D3) ELISA Kit (Cayman Chemical, Ann Arbor, MI, USA, Cat. No. 501620) according to the manufacturer’s instructions, with a detection range of 0.3–10 ng/mL. All measurements were performed in duplicate, and samples with a coefficient of variation > 15% were re-analyzed.

### 4.3. Statistical Analysis

Statistical analyses were performed using GraphPad Prism 11 (GraphPad Software, San Diego, CA, USA). Continuous variables are presented as median and interquartile range (IQR). Between-group comparisons were performed using the Mann–Whitney U test, given the non-parametric distribution of biomarker data assessed by the Shapiro–Wilk test. Rank-biserial correlation coefficients (r) were calculated as effect size measures for between-group comparisons. Associations between continuous variables were evaluated using Spearman’s rank correlation coefficient (ρ). Diagnostic performance was assessed by receiver operating characteristic (ROC) curve analysis using day 1 biomarker concentrations. The area under the ROC curve (AUC) with 95% confidence intervals (CI) was calculated using the Wilson/Brown method with 2000 bootstrap resamples. Optimal diagnostic cutoffs were determined by the Youden index (J = sensitivity + specificity − 1). Sensitivity, specificity, and cutoff values are reported for each biomarker. The available sample sizes for each biomarker are reported in the ROC analysis table. Given the exploratory nature of this study, no correction for multiple comparisons was applied. A two-sided *p* < 0.05 was considered statistically significant. A post hoc power calculation was performed for the primary day-1 ROC comparisons (G*Power 3.1, two-sided, α = 0.05). With 36 sepsis cases and 60 controls, the study had≥ 80% power to detect an AUC of ≥0.70 versus the null value of 0.50, corresponding to the effect size observed for CRP, IL-6, and cfDNA.

## Figures and Tables

**Table 1:** Receiver operating characteristic analysis of day 1 biomarkers for neonatal sepsis. S = sepsis; C = controls; Sens. = sensitivity; Spec. = specificity. Optimal cutoffs determined by Youden index.

Marker	Sepsis (n)	Control (n)	AUC (95% CI)	Sens.	Spec.	Cutoff
CRP (mg/L)	36	57	0.75 (0.66–0.85)	0.56	0.96	11.0
IL-6 (ng/L)	36	56	0.73 (0.61–0.83)	0.58	0.84	189.7
cfDNA (ng/mL)	31	48	0.72 (0.60–0.84)	0.74	0.71	653.1
MPO-DNA	29	40	0.47 (0.34–0.60)	0.97	0.20	555.0
NE-DNA	26	33	0.44 (0.29–0.59)	1.00	0.06	476.0
H3cit (ng/mL)	15	14	0.47 (0.25–0.71)	0.40	0.86	0.5

## Data Availability

Data are available from the corresponding author upon reasonable request.
